# *OsDUF3615* regulates grain size and quality traits by modulating cell proliferation and starch metabolism in rice

**DOI:** 10.3389/fpls.2026.1928838

**Published:** 2026-08-28

**Authors:** Bo Peng, Jing Qiu, Ziyue Huang, Qiaoyu Zhang, Qiang Zhao, Zhiguo Zhang, Yaqin Huang, Guanwang Shen, Wei Zhou, Jinhui Zhao, Yanfang Sun, Quanxiu Wang

**Affiliations:** 1 College of Life Sciences, Xinyang Normal University, Xinyang, China; 2 College of Horticulture, Xinyang Agriculture and Forestry University, Xinyang, China; 3 Henan Scientific Research Platform Service Center, Zhengzhou, China; 4 Henan Lingrui Pharmaceutical Company Limited, Xinyang, China; 5 College of Pharmacy, Xinyang Agriculture and Forestry University, Xinyang, China; 6 Xinyang Academy of Agricultural Science, Xinyang, China

**Keywords:** gene function, grain quality, grain size, *OsDUF3615*, rice

## Abstract

Domains of Unknown Function (DUFs) are widely distributed across diverse genomes and are increasingly recognized as important regulators of plant growth, development, and stress responses. DUF3615 is a highly conserved plant-specific protein motif; however, its biological function remains largely unknown. Previously, the gene *OsGAPC3*, a key regulator of grain quality, was isolated and functionally characterized in rice. Transcriptome analysis during the dissection of the *OsGAPC3*-mediated regulatory pathway revealed that *OsDUF3615* is significantly upregulated in *Osgapc3* mutants, suggesting its potential involvement in rice development and grain traits. In this study, we show that *OsDUF3615* is constitutively expressed in rice and encodes a nucleus-localized protein. Functional analysis demonstrated that overexpression of *OsDUF3615* significantly promotes cell proliferation and expansion in the lemma along the grain width axis, leading to increased grain width and thousand-grain weight. Moreover, *OsDUF3615* modulates grain filling dynamics and alters the accumulation of major storage compounds, including starch and free fatty acids, thereby affecting both nutritional composition and eating quality traits, such as taste value. Collectively, our findings identify *OsDUF3615* as a key regulator of rice grain development and quality formation, providing valuable genetic resources for the molecular breeding of high-quality rice varieties.

## 1 Introduction

Rice (*Oryza sativa* L.) is a staple crop critical to global food security, feeding more than half of the world’s population. In China, over two-thirds of the population relies on rice as a primary food source (Li et al., 2022; Zhao et al., 2022). With rapid socioeconomic development and improved living standards, consumer demand has shifted from quantity to quality, placing increasing emphasis on traits such as taste, nutritional value, and safety. This shift poses new challenges for the genetic improvement of rice quality. Rice quality is a multifaceted trait encompassing the perspectives of producers, processors, retailers, and consumers across the entire production chain (Peng et al., 2024; Yan et al., 2025). It is generally classified into four major components: nutritional quality, cooking and eating quality, milling quality, and appearance quality (Zhao et al., 2022). Appearance quality is mainly determined by grain shape, chalkiness, and transparency (Li et al., 2025a). Nutritional quality primarily depends on the composition of major biochemical constituents, including starch, proteins, lipids, amino acids, vitamins, and micronutrients. Among these, starch is the predominant component, consisting mainly of amylose and amylopectin (Peng et al., 2024). The composition and structural organization of starch granules in the endosperm critically influence cooking and eating quality, as well as milling and appearance traits, and may ultimately affect grain yield (She et al., 2010). Protein is the second most abundant component in rice grains and represents a major source of dietary protein worldwide, contributing approximately 15% of global protein intake. Rice protein is highly digestible and is classified into albumins, globulins, prolamins, and glutelins based on solubility (Zhou et al., 2019). Several key genes regulating protein accumulation have been identified. For example, *OsAAP6* positively regulates grain protein content (Peng et al., 2014), *OsGluA2* is involved in glutelin biosynthesis, and its mutation markedly reduces glutelin levels (Yan et al., 2019), and *OsASN1*, encoding asparagine synthetase, plays an essential role in both grain yield and protein accumulation (Lee et al., 2020). Cooking and eating quality refer to the physicochemical and sensory properties of rice during cooking, typically evaluated by amylose content, gel consistency, gelatinization temperature, and sensory taste scores (Li et al., 2024; Peng et al., 2024). These traits are among the most important determinants of consumer preference. Milling quality, another key economic trait, is commonly assessed by brown rice rate, milled rice rate, and head rice rate (Lu et al., 2025), which collectively determine processing efficiency and market value.

The evolution and functional diversification of plant gene families are key drivers of species adaptation. Domains of Unknown Function (DUFs) represent a large class of conserved protein domains that are widely distributed across diverse organisms (Bateman et al., 2010). Functional characterization of DUF-containing genes is therefore essential for elucidating the molecular basis of fundamental biological processes. Accumulating evidence indicates that DUF gene families are involved in a broad range of plant biological processes, including growth, development, and stress responses. In *Arabidopsis thaliana*, members of the AtDUF6, AtDUF246, and AtDUF579 families contribute to cell wall biosynthesis and remodeling (Ranocha et al., 2010; Stonebloom et al., 2016; Brown et al., 2011). In tomato, *SlDUF966–5* and *SlDUF966–7* enhance tolerance to cold and salt stresses (Wang et al., 2025). In rice, the DUF640 family member *TH1* positively regulates grain size and shape (Li et al., 2012; Yan et al., 2013), whereas the DUF1644 family member *OsSIDP366* plays a critical role in drought and salt stress responses (Guo et al., 2016). In maize, DUF1685 family members have been implicated in pollen development (Luo et al., 2022). Collectively, these studies highlight the diverse functional roles of DUF proteins in plant growth, development, and environmental adaptation.

Despite these advances, the biological function of *OsDUF3615* remains largely unknown. In particular, whether *OsDUF3615* is involved in rice growth and development and contributes to the regulation of grain quality has yet to be elucidated. Previously, we identified *OsAAP6* as a major quantitative trait locus (QTL) gene that positively regulates grain protein content and nutritional quality in rice (Peng et al., 2014). Further investigation of its regulatory mechanism revealed that *OsGAPC3* physically interacts with *OsAAP6* and modulates grain storage composition and quality traits by regulating *OsAAP6* expression (Peng et al., 2024). Transcriptome analysis showed that *OsDUF3615* is significantly upregulated in *OsGAPC3* mutants, while exhibiting an opposite expression pattern in *OsGAPC3*-overexpressing lines (Peng et al., 2024), suggesting that *OsDUF3615* may affect grain quality in rice.

Subsequent analyses demonstrated that *OsDUF3615* is constitutively expressed and encodes a nucleus-localized protein. Functional characterization revealed that overexpression of *OsDUF3615* increases both cell length and cell number along the grain width axis of the lemma, leading to enhanced grain width. In addition, *OsDUF3615* regulates the accumulation of major storage compounds, including starch and free fatty acids, thereby influencing multiple rice quality traits. Notably, *OsDUF3615* affects thousand-grain weight without altering yield per plant. Taken together, these findings indicate that *OsDUF3615* plays a critical role in coordinating grain development and quality formation in rice, providing valuable insights for the molecular design and breeding of high-quality rice varieties.

## 2 Materials and methods

### 2.1 Generation of *OsDUF3615* overexpression transgenic lines

The full-length cDNA of *OsDUF3615* was cloned (*OsDUF*-OX-F:5’-AATGGATCCAACTCTGT CGCCGTCG-3’, *OsDUF-OX-*R:5’- GCAGGTACCACAACCATAACATAGAGTCACAAG-3’) into the pMD18-T vector to generate the recombinant plasmid pMD18T-*OsDUF3615*, which was verified by sequencing. The validated insert was subsequently excised using *Bam* HI and *Kpn* I and ligated into the pRHVcGFP overexpression vector, yielding the construct pRHVcGFP-*OsDUF3615*. The resulting plasmid was introduced into rice (Zhonghua 11, ZH11) via *Agrobacterium tumefaciens*-mediated transformation (performed by Wuhan Tianwen Biotechnology Co., Ltd.), and transgenic lines overexpressing *OsDUF3615* were obtained.

### 2.2 Construction of subcellular localization vector

The coding sequence (CDS) of *OsDUF3615* was retrieved from the National Rice Data Center. Gene-specific primers (*OsDUF*-SL-F: 5’-CAGGGTACCCACATGAAGCTAAGTTAGC-3’, *OsDUF*-SL-R:5’-CAGTCTAGAATCGTCATCTGAATCATCC-3’) were designed using the NCBI Primer Design Tool, and the target fragment was amplified from rice cultivar Zhonghua 11 (ZH11). The amplified fragment was first cloned into the pMD18-T vector and sequence-verified. The confirmed insert was then digested with *Kpn* I and *Xba* I and ligated into the pM999–616 vector to generate the subcellular localization construct pM999-616-*OsDUF3615*.

### 2.3 RNA extraction and quantitative real-time PCR

Total RNA was extracted using TRIzol reagent (Takara) following the manufacturer’s instructions. First-strand cDNA was synthesized using the HyperScript III RT SuperMix kit (R202-02, with gDNA Remover). Quantitative real-time PCR (qRT-PCR) was performed using 2× SYBR Green Fast qPCR Master Mix (YiFeiXue Bio-Technology, China) on an ABI 7300 Real-Time PCR system. The thermal cycling conditions were as follows: 95 °C for 30 s, followed by 40 cycles of 95 °C for 15 s and 60 °C for 30 s. *β-Actin* was used as the internal reference gene, and relative gene expression levels were calculated using the 2*^−ΔΔCt^* method (Peng et al., 2024). Primers used in this study are listed in **Supplementary Table 1**. Three independent biological replicates were performed for each assay. For each biological replicate, 3 technical parallel measurements were performed to eliminate random operation errors.

### 2.4 Expression pattern analysis

Gene-specific primers for *OsDUF3615* were designed based on its coding sequence. Total RNA was extracted from various tissues of rice cultivar Zhonghua 11 (ZH11), including young roots, young stems, young leaves, roots at the heading stage, stems at the heading stage, leaves at the heading stage, and panicles at the heading stage (Peng et al., 2024). cDNA synthesis and qRT-PCR were conducted as described above. *β-Actin* was used as the internal control, and all experiments were performed with three biological replicates.

### 2.5 Subcellular localization

Rice protoplasts were isolated from 12-day-old seedlings and used for transient expression assays. The recombinant plasmid pM999-616-*OsDUF3615* and the empty vector control (pM999-GFP) were separately introduced into rice leaf sheath protoplasts. Fluorescence signals were observed using a confocal laser scanning microscope (Leica TCS SP8) as previously described (Peng et al., 2024).

### 2.6 Growth conditions and determination of grain quality traits

Rice plants were grown under field conditions at the experimental station of Xinyang Normal University with a planting density of 16.5 cm (intra-row) × 26 cm (inter-row). Standard agronomic management practices were applied in 2024 and 2025. After harvest, seeds were air-dried and stored at room temperature for at least three months before quality evaluation (Peng et al., 2024). Fully filled grains were selected for analysis of quality and yield-related traits. Protein content, amylose content, free fatty acid content, and taste value were measured using a near-infrared grain analyzer (INFRAEC Nova) (Peng et al., 2024). Gelatinization temperature was determined using the alkali spreading value method, gel consistency and grain processing quality traits (brown rice rate, milled rice rate, head rice rate) were measured according to the Chinese National Standard (GB/T 17891-1999, High-Quality Rice) (Peng et al., 2014). Milled rice was used to determine total starch content (Peng et al., 2014). Grain weights determined on samples of 200 grains were converted to 1,000-grain weights (Peng et al., 2014). All measurements were conducted with three biological replicates. All data were first subjected to Shapiro Wilk test to determine normality, and Levene test to determine homogeneity of variance. Student’s *t*-test was used for those who met the criteria; The Mann Whitney U test was used for those who were dissatisfied. For multi phenotype comparison, FDR method was used for *P*-value correction to control false positive rate.

### 2.7 Haplotype analysis

Single-nucleotide polymorphisms (SNPs) within the coding region of *OsDUF3615* were analyzed using sequence data from 533 cultivated rice accessions obtained from the Rice Variation Map Database (2023 release). Based on sequence variation, four representative haplotypes were identified for further analysis (Yang et al., 2025). The phenotypic data of grain length, grain width, grain thickness, and grain weight for 533 cultivated rice accessions were obtained from the Rice Genome Project Database (https://ricevarmap.ncpgr.cn/vars_genotype/).

## 3 Results

### 3.1 *OsDUF3615* is highly expressed in panicles and encodes a nucleus-localized protein

**Figure 1:**
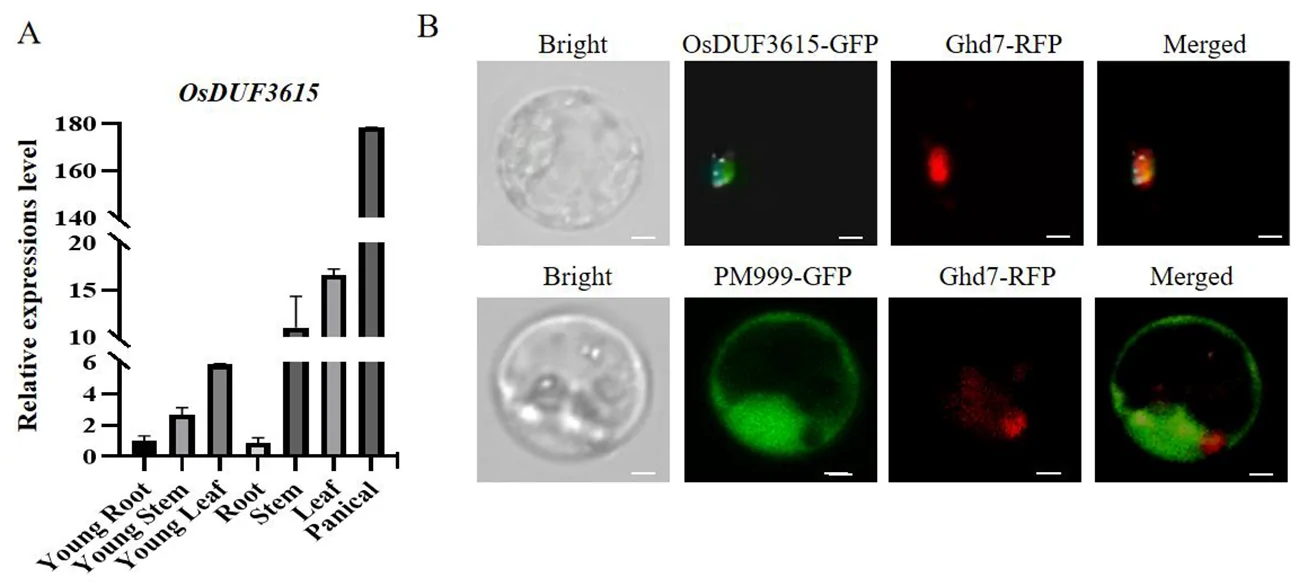
Expression pattern analysis and subcellular localization of *OsDUF3615* in rice. **(A)** Detection and analysis of the expression levels of the *OsDUF3615* gene in different rice tissues; Root, roots at the heading stage; Stem, stems at the heading stage; Leaf, leaves at the heading stage. Three independent biological replicates were performed for each assay. For each biological replicate, 3 technical parallel measurements were performed to eliminate random operation errors. **(B)** Subcellular localization of GFP. PM999-GFP is an empty vector (used as a control). Error bars represent the standard error of the mean (SEM). Co-localization of OsDUF3615 with the Ghd7 protein and subcellular localization of the empty vector PM999-GFP. Green, red, and yellow fluorescence represent the autofluorescence of OsDUF3615-GFP, Ghd7-RFP, and the merged fluorescence, respectively. Scale bars, 5 μm.

To characterize the expression pattern of *OsDUF3615*, qRT-PCR analysis was performed using RNA isolated from seven rice tissues, including young roots, young stems, young leaves, roots at the heading stage, stems at the heading stage, flag leaves, and young panicles. The results showed that *OsDUF3615* was expressed in all tested tissues, with markedly higher expression in panicles (**Figure 1A**). This pattern is consistent with expression data from the CREP database (*LOC_Os11g42850*, **Supplementary Figure 1A**), indicating that *OsDUF3615* is constitutively expressed with preferential accumulation in panicles.

To determine its subcellular localization, an *OsDUF3615*-GFP fusion construct was co-expressed with the nuclear marker *Ghd7*-RFP in rice protoplasts. The GFP control (pM999-GFP) exhibited ubiquitous fluorescence signals in multiple cellular compartments, including the plasma membrane, cytoplasm, and nucleus. In contrast, the *OsDUF3615*-GFP signal exclusively co-localized with *Ghd7*-RFP in the nucleus (**Figure 1B**), demonstrating that *OsDUF3615* encodes a nucleus-localized protein. Collectively, these results indicate that *OsDUF3615* is a constitutively expressed gene with preferential expression in panicles and nuclear localization of its encoded protein.

### 3.2 Generation and identification of *OsDUF3615* overexpression lines

A total of 24 independent transgenic lines (overexpressing *OsDUF3615*) were generated via *Agrobacterium tumefaciens*-mediated transformation, among which 22 lines (T_0_) were confirmed as positive (**Supplementary Figure 1B**). qRT-PCR analysis revealed that *OsDUF3615* transcript levels were significantly elevated in the positive overexpression (OX) lines (including *OsDUF3615*(*OX*)-1 and *OsDUF3615*(*OX*)-2) compared with the control, confirming successful overexpression (**Supplementary Figure 1C**). To further validate the effect of *OsDUF3615* overexpression transgenic, we randomly selected two lines (*OsDUF3615*(*OX*)-1 and *OsDUF3615*(*OX*)-2) and developed two corresponding families (T_1_) in the rice experimental field of Xinyang Normal University, and conducted subsequent experiments.

### 3.3 Overexpression of *OsDUF3615* promotes grain widening

**Figure 2:**
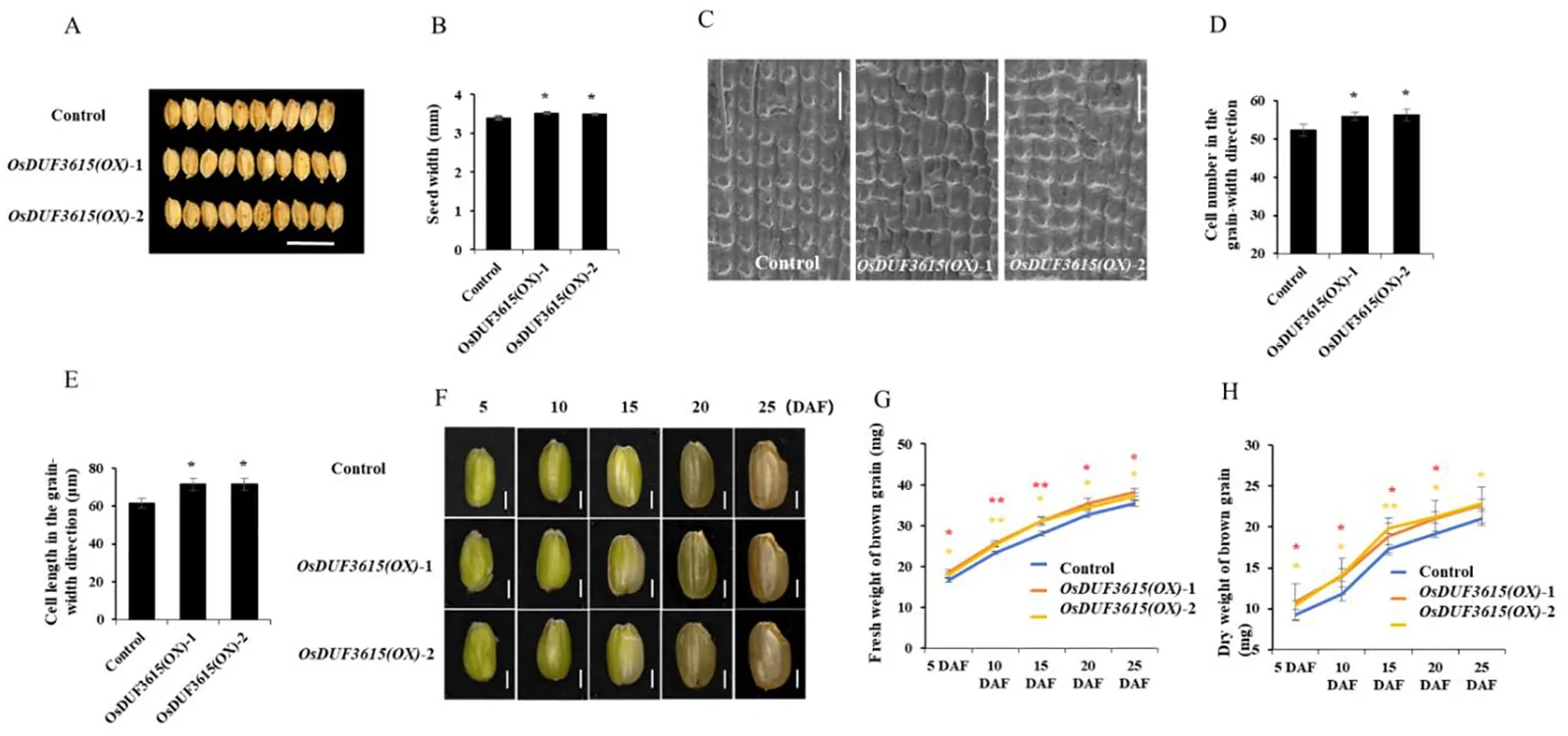
Analysis of appearance quality characteristics of rice *OsDUF3615-*OX. **(A)** *OsDUF3615* overexpression transgenic positive seed width phenotype; Scale bar, 1 cm, n=60. **(B)** Statistical analysis of *OsDUF3615* overexpression transgenic positive seed width. **(C)** Overexpression of *OsDUF3615* transgenic positive rice mature seed outer glume phenotype; Scale bars, 200 μm, n=5. **(D)** Statistical analysis of cell length in the grain width direction of *OsDUF3615* overexpressing transgenic positive rice outer husk cells. **(E)** Statistical analysis of the number of cells in the grain width direction of *OsDUF3615* overexpressing transgenic positive rice outer husk cells. **(F)** The dehulling phenotype of *OsDUF3615* overexpressing transgenic positive plants during the grain filling stage at 5, 10, 15, 20, and 25 days after flowering (DAP); scale bars, 1 mm, n=10. **(G)** Dynamic changes in fresh weight of *OsDUF3615* overexpressing transgenic positive plants during the grain filling stage. **(H)** Analysis of dynamic changes in dry weight during grain filling stage in transgenic positive plants overexpressing *OsDUF3615*. Control, overexpression of transgenic negative control. For each biological replicate, 3 technical parallel measurements were performed to eliminate random operation errors. Significant differences at the levels of ^**^P = 0.01 and ^*^P = 0.05, respectively. Significant differences were based on a two-tailed *t*-test. Error bars, standard error of the mean (SEM).

To determine whether *OsDUF3615* influences rice grain morphology, grain size parameters were analyzed in OX lines. Compared with the control, *OsDUF3615*-OX plants exhibited a significant increase in grain width, whereas grain length and thickness remained unchanged (**Figures 2A, B**; **Supplementary Figures 2A–C**). To elucidate the cellular basis underlying grain widening, spikelet hulls were examined by scanning electron microscopy. The results showed that overexpression of *OsDUF3615* significantly increased both cell length and cell number along the grain width axis, with no detectable changes along the longitudinal axis (**Figures 2C–E**; **Supplementary Figures 2D, E**). These findings indicate that *OsDUF3615* promotes grain widening by enhancing cell expansion and proliferation in the transverse direction. To further characterize grain development, fresh and dry weights of developing grains were measured at 5, 10, 15, 20, and 25 days after flowering (DAF). Both fresh and dry weights were significantly higher in *OsDUF3615*-OX lines than in the controls (**Figures 2F–H**), suggesting *OsDUF3615* enhanced grain filling. In addition, chalkiness-related traits, including chalkiness rate, chalkiness area, and chalkiness degree, were evaluated. No significant differences were observed between *OsDUF3615*-OX lines and the controls (**Supplementary Figures 2F–H**), indicating that *OsDUF3615* does not affect grain chalkiness. Collectively, these results demonstrate that overexpression of *OsDUF3615* promotes grain widening by enhancing cell proliferation and expansion during grain development, without altering grain chalkiness traits.

### 3.4 *OsDUF3615* regulates the accumulation of storage compounds and expression of starch metabolism–related genes in rice endosperm

**Figure 3:**
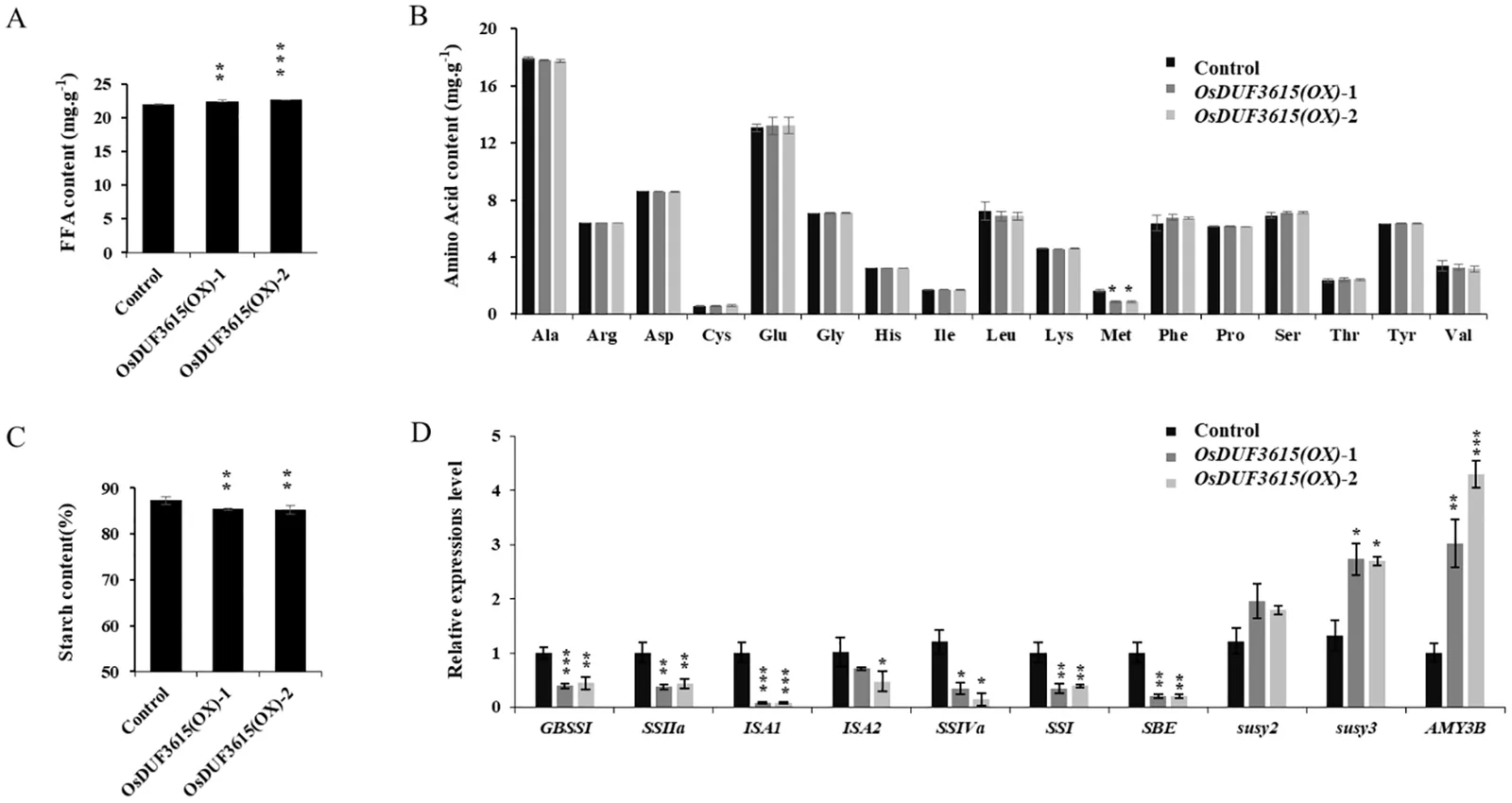
Analysis of storage substance content and starch related gene expression in *OsDUF3615* transgenic rice. **(A)** Overexpression of *OsDUF3615* transgenic rice in terms of free fatty acid content. **(B)** Overexpression of amino acid content in *OsDUF3615* transgenic rice. **(C)** Overexpression of *OsDUF3615* resulted in a decrease in total starch content in transgenic rice. All experiments were performed with three biological replicates. Each individual plant had at least 300 grains per replicate, with no less than 5 plants from the same genotype. **(D)** Detection and analysis of expression levels of starch metabolism related genes in transgenic rice grains with overexpression of *OsDUF3615* during grain filling stage; The data came from 3 independent biological replicates, with 3 technical replicates set for each sample. Control, overexpression of transgenic negative control. Significant differences at the levels of ^***^P = 0.001, ^**^P = 0.01 and ^*^P = 0.05, respectively. Significant differences were based on a two-tailed *t*-test. Error bars, standard error of the mean (SEM).

To evaluate the effects of *OsDUF3615* on storage compound accumulation, major grain components were quantified in overexpression (OX) lines using a near-infrared grain analyzer. The results showed that *OsDUF3615* overexpression significantly increased free fatty acid content, while total starch and methionine contents were markedly reduced. In contrast, no significant changes were observed in total protein, total amino acids, or essential amino acids (**Figures 3A–C**; **Supplementary Figures 3A–C**). These findings indicate that *OsDUF3615* modulates the accumulation of key storage compounds, particularly starch and lipids, in rice grains. Given the pronounced reduction in starch content, the expression of genes involved in starch metabolism was further examined at 10 days after fertilization (DAF). qRT-PCR analysis revealed that multiple starch biosynthesis genes, including *GBSSI*, *SSI*, *SSIIa*, *SSIVa*, and *SBE*, were significantly downregulated in *OsDUF3615*-OX lines. In contrast, the starch degradation–related gene *AMY3B* was significantly upregulated (**Figure 3D**; **Supplementary Table 1**). These expression patterns are consistent with the observed decrease in starch accumulation, suggesting that *OsDUF3615* negatively regulates starch biosynthesis while promoting starch degradation during grain filling.

### 3.5 Overexpression of *OsDUF3615* compromises eating quality but does not affect milling quality

**Figure 4:**
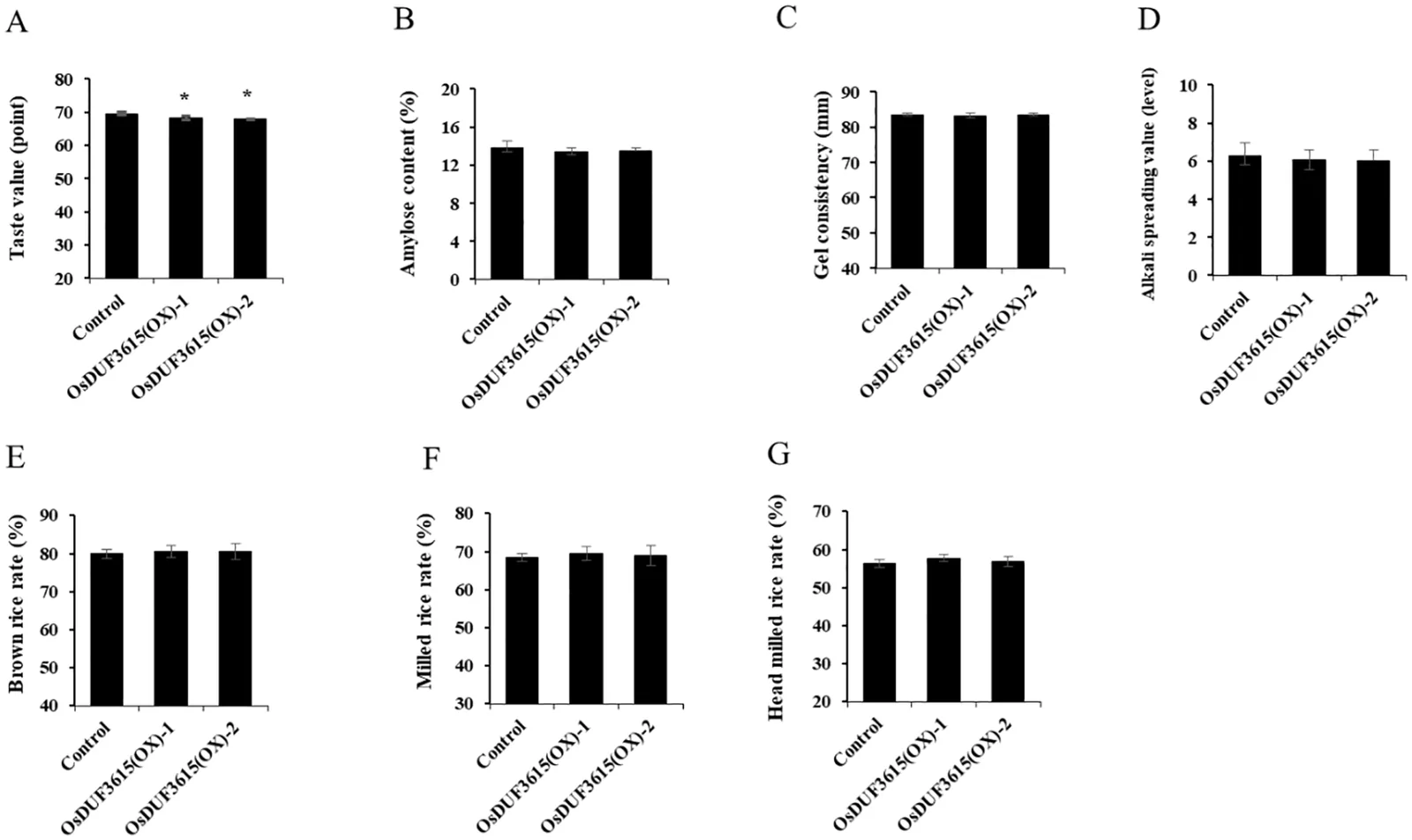
Detection and analysis of eating and milling quality traits of *OsDUF3615* transgenic rice. **(A)** Taste value of *OsDUF3615* transgenic rice. **(B)** Amylose content of *OsDUF3615* transgenic rice. **(C)** Gel consistency of *OsDUF3615* transgenic rice. **(D)** Gelatinization temperature of *OsDUF3615* transgenic rice. **(E)** Brown rice rate of *OsDUF3615* transgenic rice. **(F)** Milled rice rate of *OsDUF3615* transgenic rice. **(G)** Head milled rice rate of *OsDUF3615* transgenic rice. Control, overexpression of transgenic negative control. All experiments were performed with three biological replicates. Each individual plant had at least 300 grains per replicate, with no less than 5 plants from the same genotype. Significant differences at the levels of ^*^P = 0.05, respectively. Significant differences were based on a two-tailed *t*-test. Error bars, standard error of the mean (SEM).

To assess the impact of *OsDUF3615* on eating quality, key parameters including amylose content, gel consistency, gelatinization temperature, and taste value were evaluated in overexpression (OX) lines. The results showed that *OsDUF3615* overexpression significantly reduced taste value and amylopectin content, whereas no significant differences were observed in amylose content, gel consistency, or gelatinization temperature compared with the control (**Figures 4A–D**; **Supplementary Figure 3D**). These results indicate that *OsDUF3615* negatively affects eating quality without altering the major physicochemical properties of starch. To further determine its effect on processing performance, milling-related traits were analyzed. No significant differences were detected in brown rice rate, milled rice rate, or head rice rate between *OsDUF3615*-OX lines and the control (**Figures 4E–G**), indicating that *OsDUF3615* does not influence milling quality. Collectively, these results demonstrate that while *OsDUF3615* overexpression compromises eating quality, it has no detectable impact on grain milling quality.

### 3.6 Effects of *OsDUF3615* on yield-related traits

**Figure 5:**
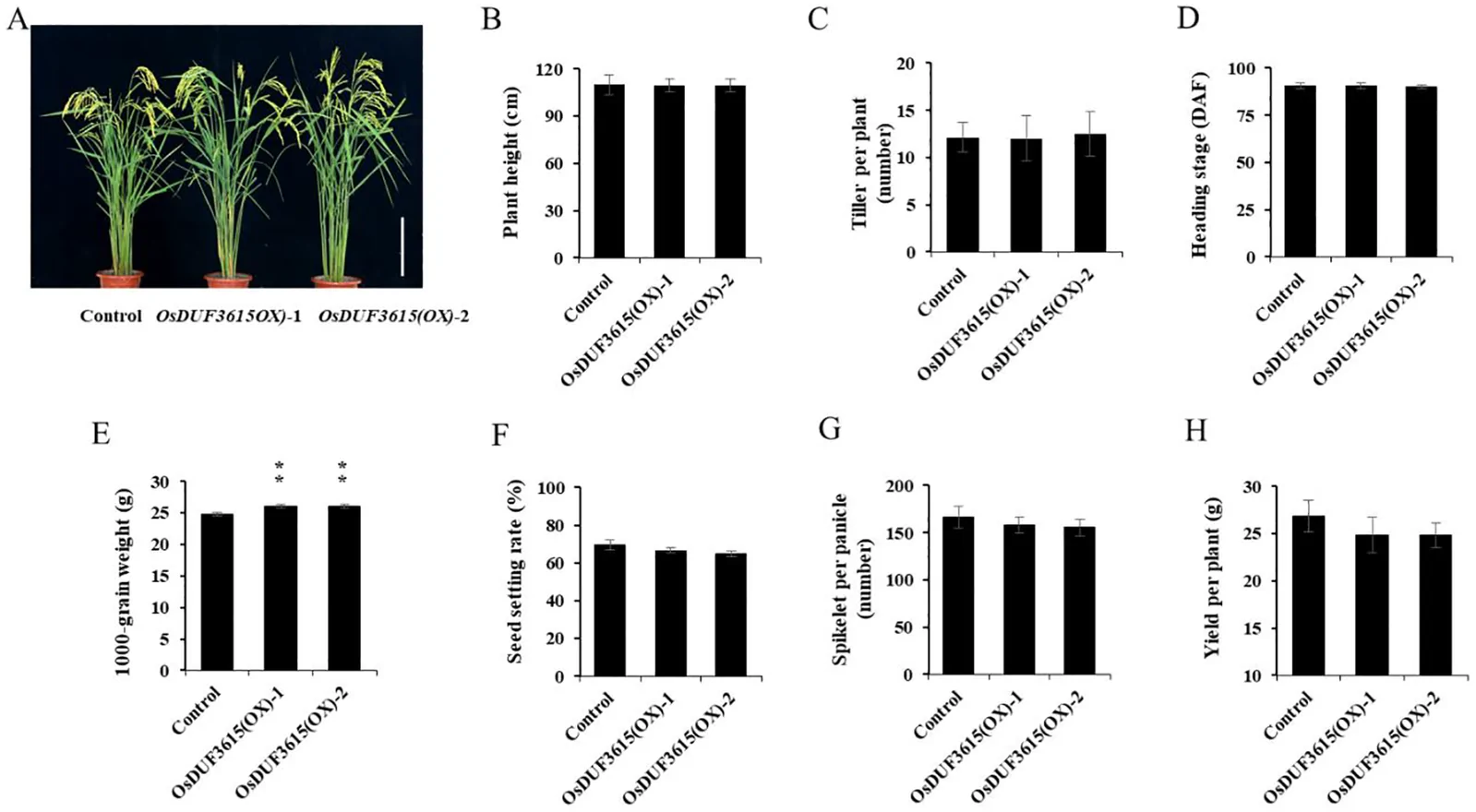
Analysis of agronomic and yield traits detection of *OsDUF3615* transgenic plants. **(A)** Phenotypic observation of transgenic plants overexpressing *OsDUF3615*, scale bar: 30 cm. **(B)** Plant height. **(C)** Tiller per plant. **(D)** Heading stage. **(E)** Thousand grain weight. **(F)** Seed setting rate. **(G)** Spikelet per panicle. **(H)** Yield per plant. Control, overexpression of transgenic negative control, n=60. All experiments were performed with three biological replicates. Significant differences at the levels of ^**^P = 0.01. Significant differences were based on a two-tailed *t*-test. Error bars, standard error of the mean (SEM).

Yield-related traits were evaluated in *OsDUF3615* overexpression (OX) lines. The results showed that thousand-grain weight was significantly increased in *OsDUF3615*-OX plants compared with the control. However, no significant differences were observed in spikelet number per panicle, seed-setting rate, plant height, tiller number, heading date, or yield per plant (**Figure 5**). These results indicate that although *OsDUF3615* overexpression enhances grain weight, it does not affect the yield per plant.

### 3.7 Haplotype analysis of *OsDUF3615* reveals associations with grain size variation

**Figure 6:**
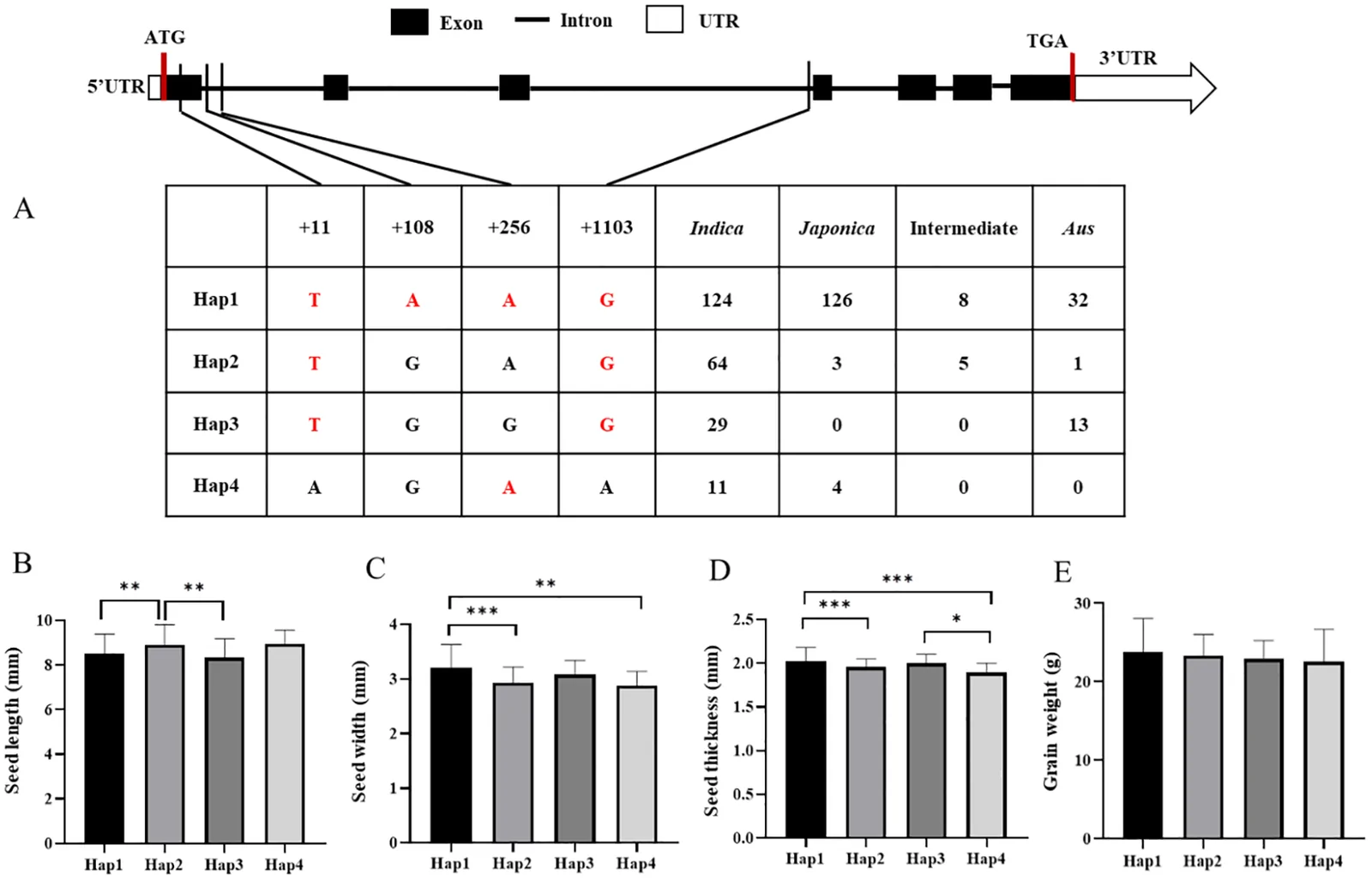
Haplotype analysis of *OsDUF3615* and grain related traits. **(A)** Haplotype identification of *OsDUF3615* coding region. **(B)** Comparison of grain length among four haplotypes. **(C)** Comparison of grain width among four haplotypes. **(D)** Comparison of grain thickness among four haplotypes. **(E)** Comparison of thousand grain weight of four haplotypes. The significant difference is based on Tukey’s multiple comparisons, significant differences were based on a two-tailed *t*-test, ^***^P<0.001, ^**^P<0.01, ^*^P < 0.05. Error bars, standard error of the mean (SEM).

To assess the potential impact of natural variation in *OsDUF3615* on grain traits, haplotype analysis was performed using sequence data from 533 cultivated rice accessions obtained from the Rice Variation Map database (https://ricevarmap.ncpgr.cn/vars_genotype/). Four single nucleotide polymorphisms (SNPs) were identified in the coding region of *OsDUF3615*, which defined four major haplotypes (Hap1–Hap4 included 420 rice accesses, the remaining 113 rice accessions belonged to multiple low-frequency rare haplotypes, and had not natural population representativeness and were not suitable for statistical comparison of grain phenotypes). Haplotype distribution analysis showed that Hap1 was the most prevalent and was present in both *indica* and *japonica* subspecies, with a slightly higher frequency in *japonica* rice (included Zhonghua 11, ZH11). In contrast, Hap2, Hap3, and Hap4 were predominantly detected in *indica* rice (**Figure 6A**). Phenotypic comparison among haplotypes revealed significant differences in grain size traits. Specifically, significant differences in grain length were observed between Hap1 vs Hap2 and Hap2 vs Hap3. Grain width also differed significantly between Hap1 vs Hap2 and Hap1 vs Hap4, as well as between Hap3 vs Hap4. However, no significant differences were detected in thousand-grain weight among the four haplotypes (**Figures 6B–E**). Collectively, these results indicate that natural allelic variation in *OsDUF3615* is associated with grain size variation, suggesting its potential contribution to the genetic regulation of rice grain morphology in different rice populations.

## 4 Discussion

In monocotyledonous grasses, glyceraldehyde-3-phosphate dehydrogenase C (GAPC) plays essential roles in plant growth, development, and stress responses (Kim et al., 2022). In rice, mutation of *OsGAPC3* significantly affects grain quality traits and improves nutritional properties. During the functional dissection of *OsGAPC3*, *OsDUF3615* was identified as an important responsive gene, showing strong upregulation in *Osgapc3* mutants and an opposite expression pattern in *OsGAPC3*-overexpressing plants (Peng et al., 2024). Domains of unknown function (DUFs) represent a large class of conserved proteins that are increasingly recognized as key regulators of plant growth and development (Wang et al., 2025; Li et al., 2012; Yan et al., 2013). However, the biological function of *OsDUF3615* in rice remained unclear prior to this study. Here, we demonstrate that *OsDUF3615* is a constitutively expressed gene encoding a nucleus-localized protein, suggesting its potential involvement in transcriptional or regulatory processes.

**Figure 7:**
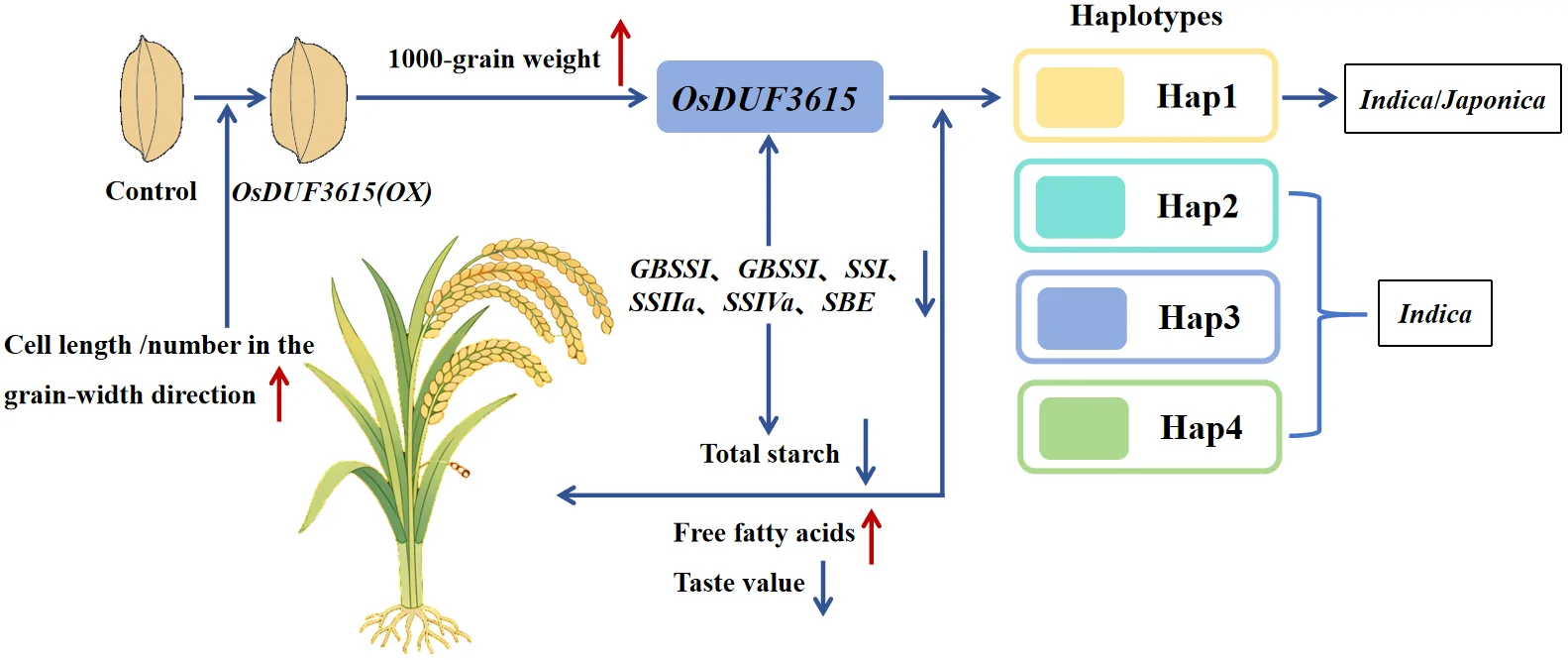
Molecular model of *OsDUF3615* regulating rice grain type and quality in rice. *OsDU3615* is highly expressed in the panicle and localized in the nucleus. Overexpression of *OsDU3615* can increase rice grain width and thousand grain weight. By regulating the expression of genes related to starch synthesis and degradation, it changes the starch and fatty acid content of rice, ultimately affecting the taste and quality of rice. At the same time, this gene naturally affects the size difference between *indica* and *japonica* rice.

Grain morphology is a primary determinant of rice appearance quality (Li et al., 2025c). Our results show that overexpression of *OsDUF3615* significantly increases grain width without affecting grain length or thickness. Cytological analysis further revealed that this phenotype is associated with increased cell length and cell number in the transverse direction of the lemma, indicating that *OsDUF3615* regulates grain width through coordinated control of cell proliferation and expansion (**Figure 7**). Moreover, dynamic measurements during grain filling (5–25 DAF) showed enhanced grain development in *OsDUF3615* overexpression lines, supporting its role in promoting grain widening during endosperm development. Grain nutritional quality is largely determined by the accumulation of major storage compounds, particularly starch and protein (Peng et al., 2014; Singh et al., 2015). In this study, *OsDUF3615* overexpression significantly altered grain metabolic composition by reducing total starch and methionine content while increasing free fatty acid levels (**Figures 3A–C**; **Supplementary Figure 3D**). Consistently, starch biosynthesis-related genes were markedly downregulated, whereas the starch degradation gene *AMY3B* was upregulated in developing grains (**Supplementary Figure 3D**). These results indicate that *OsDUF3615* also regulates starch biosynthesis while promoting starch degradation, thereby reshaping carbon allocation during grain filling. As the OsDUF3615 localized in cell nucleus, it is possible that OsDUF3615 directly binds to genes related to sucrose synthesis through protein-protein interactions, inhibiting their transcriptional activity in the endosperm. Alternatively, *OsDUF3615* may also target and regulate the transcriptional activity of multiple genes related to starch and sucrose metabolism, thereby influencing the metabolic pathway of endosperm starch synthesis. The related molecular regulatory mechanisms require further investigation.

Eating quality is a key trait determining consumer preference and is mainly evaluated by amylose content, gel consistency, gelatinization temperature, and taste value (Li et al., 2025b). Although most physicochemical parameters were not significantly affected, *OsDUF3615* overexpression significantly reduced taste value, suggesting a negative effect on eating quality that may be associated with altered starch composition and metabolism. Yield is a complex trait composed of multiple agronomic components, including spikelet number, seed setting rate, tiller number, and thousand-grain weight (Guo et al., 2025). Here, we found that *OsDUF3615* significantly increased thousand-grain weight but did not affect the yield per plant, indicating that enhanced grain size does not translate into increased yield at the whole-plant level, possibly due to compensation among yield components. Simultaneously, *OsDUF3615* participates in the regulation of major storage compound accumulation, including starch and free fatty acids, thereby influencing both nutritional composition and cooking and eating quality of rice grains. The aforementioned experimental results were obtained through the analysis of field agronomic traits and grain quality testing of transgenic plants overexpressing *OsDUF3615*. In the later stage, it is necessary to further generate mutants or gene knockout materials corresponding to *OsDUF3615* for reverse genetic verification.

Natural allelic variation plays a crucial role in the diversification of agronomic traits (Yang et al., 2019). Haplotype analysis revealed four major *OsDUF3615* haplotypes, with Hap1 widely distributed across both *indica* and *japonica* subspecies, while Hap2–Hap4 were mainly enriched in *indica* rice. Significant differences in grain length and width were observed among haplotypes, further supporting the association between *OsDUF3615* allelic variation and grain morphology. Although no significant differences in thousand-grain weight were detected, these results indicate that *OsDUF3615* is closely associated with natural variation in grain size. Grain size is a crucial factor influencing the grain appearance quality of rice. There are significant variations in the demand for rice grain types across different regions. The aforementioned research results indicate that *OsDUF3615* contributes to increasing the grain width and weight of rice seeds, and influences the grain nutritional and cooking quality of the rice. This provides valuable functional gene resources for molecular marker-assisted selection in the development of new high-quality rice varieties.

## 5 Conclusions

*OsDUF3615* is a constitutively expressed gene encoding a nucleus-localized protein in rice. Functional characterization demonstrates that overexpression of *OsDUF3615* enhances grain width by promoting both cell proliferation and expansion in the lemma, and alters grain developmental dynamics during the filling stage, leading to increased grain width and thousand-grain weight, while grain yield per plant remains unaffected. In addition, *OsDUF3615* participates in the regulation of major storage compound accumulation, including starch and free fatty acids, thereby influencing both nutritional composition and eating quality of rice grains. Collectively, *OsDUF3615* is a key regulator of multiple grain quality traits and grain development processes, providing valuable genetic resources for the molecular breeding of high-quality rice varieties.

## Data Availability

The original contributions presented in the study are included in the article/**Supplementary Material**. Further inquiries can be directed to the corresponding authors.
