# Supplementary material for: OsDUF3615 regulates grain size and quality traits by modulating cell proliferation and starch metabolism in rice
Source: Front Plant Sci. 2026 Aug 28;17:1928838. doi: 10.3389/fpls.2026.1928838 (PMC13564115; doi:10.3389/fpls.2026.1928838)
Supplement: Supplementary file 1 [file Table1.docx]

**Figure S1** *OsDUF3615* expression level analysis and expression level detection. (A) Expression level analysis of *OsDUF3615* (*LOC_S11g42850*) in different tissues of CREP database (http://crep.ncpgr.cn/crep-cgi/query_by_tree.cgi). (B) Positive identification of transgenic lines overexpressing *OsDUF3615* in rice (T_0_). (C) Detection of gene expression levels in *OsDUF3615* overexpressing transgenic lines, n=24. Control: overexpression transgenic negative control. Significant differences at the levels of ^***^*P*=0.001. Significant differences were based on a two-tailed *t*-test. Error bars, standard error of the mean (SEM).

**Figure S2** Analysis of appearance quality characteristics of rice *OsDUF3615*. (A) *OsDUF3615* overexpression transgenic rice grain length phenotype; Scale, 1 cm, n=60. (B) *OsDUF3615* overexpression transgenic grain length statistics. (C) Overexpression of *OsDUF3615* in transgenic rice grain thickness statistics. (D) Statistical analysis of cell length in the grain length direction of *OsDUF3615* overexpressing transgenic positive rice outer husk cells. (E) Statistical analysis of the number of cells in the grain length direction of *OsDUF3615* overexpressing transgenic positive rice outer husk cells. (F) Statistics of chalkiness rate in transgenic rice overexpressing *OsDUF3615*. (G) Statistics of chalkiness area in transgenic rice overexpressing *OsDUF3615*. (H) Statistics of chalkiness degree in transgenic rice overexpressing *OsDUF3615*. Control: overexpression transgenic negative control. All experiments were performed with three biological replicates. Each individual plant had at least 300 grains per replicate, with no less than 5 plants from the same genotype. Error bars, standard error of the mean (SEM).

**Figure S3** Analysis of nutritional quality characteristics of rice *OsDUF3615*. (A) Detection and analysis of the essential amino acid content in *OsDUF3615* overexpressing transgenic grains. (B) Detection and analysis of the total amino acid content in *OsDUF3615* overexpressing transgenic grains. (C) Detection and analysis of the protein content in *OsDUF3615* overexpressing transgenic grains. (D) Analysis of the amylopectin content in *OsDUF3615* overexpressing transgenic grains. Control: overexpression of transgenic negative control. All experiments were performed with three biological replicates. Each individual plant had at least 300 grains per replicate, with no less than 5 plants from the same genotype. Significant differences at the levels of ^**^*P*=0.01. Significant difference based on two tailed *t*-test. Error bars, standard error of the mean (SEM).

**Table S1** Primer sequences for starch related genes

| Gene | Primer sequence (5'to3') | Amplicon size (bp) |
| --- | --- | --- |
| *GBSSI-F* | TCCGAGAGGTTCAGGTCATC | 143 |
| *GBSSI-R* | ATGAGCTCCTCGGCGTAGTA |  |
| *SSI-F* | TCATGGATGTGAAGGAGCAA | 119 |
| *SSI-R* | TGGCAGTGAACCACAAACAT |  |
| *SSIIa-F* | GATCGACCAGGATGACGATT | 226 |
| *SSIIa-R* | GGGTAAAGCACCTGCAACAT |  |
| *SBE-F* | GGCATTGCACTCCAAAAGAT | 132 |
| *SBE-R* | GCTCCAGTTGTTGCCTTCTC |  |
| *Susy3-F* | CATGTACCCCCTGCTCAACT | 319 |
| *Susy3-R* | GTCAGCTGTAATGCCTGCAA |  |
| *SSIVa-F* | GGGAGCGGCTCAAACATAAA | 237 |
| *SSIVa-R* | CCGTGCACTGACTGCAAAAT |  |
| *ISA2-F* | TAGAGGTCCTCTTGGAGG | 170 |
| *ISA2-R* | AATCAGCTTCTGAGTCACCG |  |
| *SBEIIa-F* | GCCAATGCCAGGAAGATGA | 127 |
| *SBEIIa-R* | GCGCAACATAGGATGGGTTT |  |
| *ISA1-F* | TGCTCAGCTACTCCTCCATCATC | 132 |
| *ISA1-R* | AGGACCGCACAACTTCAACATA |  |
| *β-actin-F* | TGCTATGTACGTCGCCATCCAG | 199 |
| *β-actin-R* | AATGAGTAACCACGCTCCGTCA |  |
| *OsDUF3615-F* | AGGAATACCGCAAGACGCAT | 459 |
| *OsDUF3615-R* | TGAACTACGTCCACGGAAGC |  |
